# Alleles Causing Resistance to Isoxaben and Flupoxam Highlight the Significance of Transmembrane Domains for CESA Protein Function

**DOI:** 10.3389/fpls.2018.01152

**Published:** 2018-08-24

**Authors:** Isaac Shim, Robert Law, Zachary Kileeg, Patricia Stronghill, Julian G. B. Northey, Janice L. Strap, Dario T. Bonetta

**Affiliations:** ^1^Faculty of Science, University of Ontario Institute of Technology, Oshawa, ON, Canada; ^2^Department of Biological Sciences, University of Toronto Scarborough Campus, Toronto, ON, Canada

**Keywords:** CESA, isoxaben, flupoxam, *Arabidopsis*, cellulose crystallinity

## Abstract

The cellulose synthase (CESA) proteins in *Arabidopsis* play an essential role in the production of cellulose in the cell walls. Herbicides such as isoxaben and flupoxam specifically target this production process and are prominent cellulose biosynthesis inhibitors (CBIs). Forward genetic screens in *Arabidopsis* revealed that mutations that can result in varying degrees of resistance to either isoxaben or flupoxam CBI can be attributed to single amino acid substitutions in primary wall CESAs. Missense mutations were almost exclusively present in the predicted transmembrane regions of CESA1, CESA3, and CESA6. Resistance to isoxaben was also conferred by modification to the catalytic residues of CESA3. This resulted in cellulose deficient phenotypes characterized by reduced crystallinity and dwarfism. However, mapping of mutations to the transmembrane regions also lead to growth phenotypes and altered cellulose crystallinity phenotypes. These results provide further genetic evidence supporting the involvement of CESA transmembrane regions in cellulose biosynthesis.

## Introduction

Plant cell walls are a complex array of networks that are primarily made up of polysaccharides. These structures ultimately determine the shape and function of plant cells and respond to changing developmental and environmental cues to alter their composition and architecture. A cell wall polymer that stands out, because of its abundance and its mechanical strength, is cellulose. This macromolecule is composed of hydrogen bonded ß-(1,4)-D-glucose, which vary in length and angle, affecting the relative crystallinity, and strength, of the cellulose (Kumar and Turner, 2015). Ultimately, cellulose is complexed, through cross-linking, with more soluble matrix polysaccharides like hemicelluloses and pectins, and together these polymers impart the features that make the wall a semipermeable, dynamic structure (Somerville, 2006; Lei et al., 2012; McFarlane et al., 2014).

Cellulose is synthesized at the plasma membrane and is associated with cellulose synthase complexes (CSCs) that can be visualized in freeze-fracture experiments as globular “rosettes” with sixfold symmetry (Mueller and Brown, 1980; Haigler and Brown, 1986; Kimura et al., 1999). Although the true nature of these globules is not known, there is a consensus that they contain cellulose synthase subunits (Delmer, 1999; Somerville, 2006; Lei et al., 2012; McFarlane et al., 2014). Most models predict that each globule contains at least three different cellulose synthase subunits (Kennedy et al., 2007; Fernandes et al., 2011; Newman et al., 2013; Thomas et al., 2013).

In contrast to bacterial cellulose biosynthesis, it has been difficult to assay cellulose synthase activity in plants *in vitro*. Instead, plant cellulose synthase (CESA) activity has been determined via genetic studies which have provided an *in vivo* link between cellulose deposition in the wall and the *CESA* genes (Arioli et al., 1998; Taylor et al., 1999; Fagard et al., 2000). Cellulose synthase proteins are β-glycosyltransferase (GT2) enzymes that are characterized by eight transmembrane domains and a conserved cytosolic substrate binding and catalytic motif (D, D, D, and QxxRW) positioned between the second and third transmembrane domains. A current model, which is supported by the crystal structure of the BcsA and BcsB proteins from *Rhodobacter sphaeroides*, predicts that the first D, D residues coordinate uridine diphosphate (UDP) glucose and the third provides the catalytic base for glucan extension, while the QxxRW residues act as a binding site for the terminal glucan residues of the chain (Morgan et al., 2013; Omadjela et al., 2013). A similar mechanism, based on computer modeling, has been proposed for plants (Sethaphong et al., 2013). Apart from the conserved catalytic motif, plant CESAs also contains an N-terminal zinc-finger domain and the so-called plant-conserved region (P-CR) and class-specific region (CSR) located between the second and third transmembrane domains. The exact significance of these domains has yet to be fully clarified, however, an increasing number of studies have been aimed at addressing how they might function in the CSC (Kurek et al., 2002; Sethaphong et al., 2013; Olek et al., 2014; Vandavasi et al., 2016).

In *Arabidopsis*, there are 10 *CESA* paralogues which can be subdivided into primary cell wall cellulose synthesis (*CESAs* 1, 2, 3, 5, 6, and 9) requirements or secondary cell wall cellulose (*CESAs* 4, 7, and 8) requirements. Disruption of *CESA1* and *CESA3* function leads to defects in plant growth, ranging from mild to more severe defects typified by the loss of anisotropic growth, ectopic lignification, and stunted growth (Arioli et al., 1998; Scheible et al., 2001; Cano-Delgado et al., 2003) or gametophytic lethality (Persson et al., 2007). Disruption of other primary cell wall CESAs does not lead to lethal phenotypes unless multiple ones are dysfunctional indicating that they are redundant (Desprez et al., 2007; Persson et al., 2007). An outstanding challenge in understanding cellulose biosynthesis in plants continues to be in isolating active CSCs from either plants or heterologous systems. This has limited the ability to conduct in-depth structural analysis of the CSC. However, alternative approaches that make use of cellulose biosynthesis inhibitors (CBIs) have provided useful information reflecting on how CSCs might behave *in vivo* [for review see (Brabham and Debolt, 2012; Tateno et al., 2016)]. CBIs have, for example, been used to perturb the dynamics of CSCs *in vivo*, which are tracked by tagging CESAs in the primary wall (usually CESA3 or CESA6) with a fluorescent reporter protein (Paredez et al., 2006; DeBolt et al., 2007; Bischoff et al., 2009; Gutierrez et al., 2009; Harris et al., 2012; Worden et al., 2015). The use of fluorescently tagged CSCs has been used to characterize a group of CBIs, which cause CSCs to clear from the plasma membrane and accumulate in cytosolic vesicles. These include isoxaben (Paredez et al., 2006), AE F150944 (Gutierrez et al., 2009), quinoxyphen (Harris et al., 2012), CGA 325′615 (Crowell et al., 2009), thaxtomin A (Bischoff et al., 2010), CESTRIN (Worden et al., 2015), and acetobixan (Xia et al., 2014). Among these, isoxaben (Heim et al., 1989; Scheible et al., 2001; Desprez et al., 2002; Sethaphong et al., 2013) and quinoxyphen (Harris et al., 2012; Sethaphong et al., 2013) have also been used in conjunction with forward genetics in *Arabidopsis* to select for resistance alleles. Related compounds, such as the triazofenamide (Heim et al., 1998; García-Angulo et al., 2012) and its derivative, such as flupoxam (Hoffman and Vaughn, 1996; Sabba and Vaughn, 1999; Vaughn and Turley, 2001), which lead to a similar cellulose-depletion syndrome in plants, have not been tested using fluorescently tagged CESAs.

Mutations conferring resistance to isoxaben (*ixr*) map to the *CESA3 (ixr1)* or *CESA6* (*ixr2*) gene (Scheible et al., 2001). In CESA3, the G998D substitution in *ixr1-1* is located at the start of the predicted eighth transmembrane domain (TMD8) of the protein, while the T942I substitution in *ixr1*-*2* is located in an extracellular loop between the putative TMD7 and TMD8 (Scheible et al., 2001). In CESA6, resistance to isoxaben is caused by an R1064W substitution located in a predicted soluble C-terminal tail (Desprez et al., 2002). An interesting feature of these mutations is the lack of remarkable phenotypes in the absence of the herbicide. An exception is a more recently isolated allele, *ixr1-6* (Sethaphong et al., 2013), which leads to a S377F substitution located close to a conserved aspartate in the predicted catalytic region of the protein, resulting in a decreased cellulose content, cellulose crystallinity, as well as plant height. Since there is no effect on uptake or detoxification of the herbicide as a consequence of these mutations, it has been interpreted to mean that the substitutions are located in the herbicide’s targets (Scheible et al., 2001; Desprez et al., 2002). Furthermore, the feature that heterozygotes show intermediate resistance to the herbicide suggests that the mutant CESA subunits form mixed rosettes with wild-type CESAs (Scheible et al., 2001). The fact that *ixr* alleles are limited to *CESA3* and *CESA6* suggests that not all CESAs are sensitive to isoxaben and that the two CESA isoforms interact in a manner that is disrupted by the herbicide.

A mutation conferring resistance to the herbicide quinoxyphen (*aegeus*) maps to CESA1 and leads to the substitution A903V which is located in the putative TMD4 of the protein (Harris et al., 2012). As a consequence, this mutation leads to decreased cellulose crystallinity, increased saccharification as well as increased CSC mobility, indicating that the rate of polymerization is increased (Harris et al., 2012). Forward genetic screening for CBI-resistance has also been applied to the phytotoxin, thaxtomin A, leading to the identification of the *TXR1* (*THAXTOMIN RESISTANCE-1*) gene, which encodes a mitochondrial inner membrane protein, PAM16 (Scheible et al., 2003; Huang et al., 2013). At this point it is ambiguous how alleles of *TXR1*/*PAM16* might lead to thaxtomin A resistance.

In an effort to gain insights into the mode of action of isoxaben and flupoxam, we conducted forward genetic screens aimed at identifying resistance alleles of these two CBIs. Using an approach similar to Heim et al. (1989), we screened at concentrations that were, however, substantially lower than that used in earlier screens to identify additional alleles. To this end, we had successfully identified five *isoxaben resistance* (*ixr*) and seven *flupoxam resistance* (*fxr*) alleles. These alleles conferred varying degrees of CBI-resistance and were exclusively located in the *CESA1, CESA3*, and *CESA6* genes. The mutations were predominantly clustered around the C-terminal regions of the CESA proteins and caused differences in cellulose crystallinity and saccharification.

## Materials and Methods

### Plant Material and Growth Conditions

All *Arabidopsis* lines were of the Landsberg erecta (Ler) or Columbia (Col-0) ecotypes and were generated in this study with the exception of *ixr1-1, ixr1-2*, and *ixr2-1* which were previously described (Heim et al., 1989, 1990; Scheible et al., 2001; Desprez et al., 2002). The *ixr1-1, ixr1-2, and ixr2-1* seeds were obtained from the *Arabidopsis* Biological Resource Center at Ohio State University. Seedlings were germinated and grown under continuous light (200 μE/m^2^/s) or kept in the dark at 21°C on 0.8% agar plates containing 2 g/L of Murashige and Skoog (0.5× MS) mineral salts (Sigma-Aldrich, St. Louis, MO, United States). Alternatively, plants were grown using mixture of 70% sphagnum peat, 15% perlite, and 15% vermiculite in growth chambers maintaining the following conditions: 21°C under long-day conditions (16 h light/8 h dark) at a light intensity of 200 μE/m^2^/s. Flupoxam was a kind gift from Kureha Chemical Industry Co., Iwaki City, Japan and isoxaben was obtained from Sigma-Aldrich. MS-Agar was supplemented from a 100 μM stock of the herbicide dissolved in anhydrous ethanol.

### EMS Mutagenesis and Screening

Forty-five thousand Ler seeds were mutagenized by treatment with 0.3% ethylmethane sulfonate (EMS) for 16 h at room temperature. These were then extensively washed with water over the course of 10 h. The EMS-treated seeds were sown to soil to generate an M2 population exceeding 1 million seeds. Five hundred thousand of the M2 seeds were screened on isoxaben and another 500,000 on flupoxam. To do this, the seeds were germinated on 0.8% agar plates containing 0.5× MS salts with either 20 nM isoxaben or flupoxam. Resistant mutants were isolated and transferred to MS and allowed to recover for 3 days. Plants were propagated and reselected to confirm that the isoxaben or flupoxam resistance was heritable. Plants were tested on varying concentrations of inhibitor (1–1000 nM) by growing seedlings vertically on solid media for 5 days and measuring root lengths (*n* ≥ 100). To determine the dominance relationship of the *ixr* or *fxr* alleles to wild type, resistant plants were backcrossed to Ler plants to generate an F2 population for each of the mutants. These were then tested on varying concentrations of inhibitor (*n* ≥ 300) to determine segregation ratios of resistance to sensitive plants.

### Plant Measurements

Plant height measurements were determined by growing plants in soil for 45 days on a 16 h light/8 h dark cycle (*n* = 6). The diameters of rosettes were determined by growing plants in soil under the same conditions and the diameter of the rosettes measured when the emerging inflorescence became visible (*n* = 6). Diameters were measured as the distance between the far edges of rosette leaves. Hypocotyl lengths were measured using seedlings that had been grown for 5–7 days in the dark on 0.5× MS with or without 10 nM CBI (*n* ≥ 20) or supplemented with 4.5% sucrose.

### DNA Purification, DNA Sequencing, and Mutant Genotyping

Leaf tissue was ground in 150 μl of cetyltrimethylammonium bromide (CTAB) using a drill-press with a homogenizing bit in a sterile eppendorf tube. CTAB buffer consisted of 100 mM Tris buffer, pH 8.0, 1.4 M NaCl, 20 mM EDTA, and 2% w/v CTAB (adapted from Murray and Thompson, 1980). The homogenizing bit was then rinsed with another 150 μl of CTAB to clear left over tissue debris into the eppendorf tube. The tissue suspension was then incubated for 1 h at 65°C. Following incubation, an equal volume of chloroform was added and the solution was vortexed thoroughly and centrifuged for 3 min at 20,000 *g*. The aqueous phase was extracted and an equal volume of 2-propanol was added and the tube was inverted several times to precipitate DNA. The tubes were then centrifuged for 10 min at 20,000 *g*. The DNA pellet was washed with 70% ethanol, air dried and dissolved in sterile 10 mM Tris buffer pH, 8.0. The isolated genomic DNA of the selected mutant lines were used as templates for polymerase chain reaction (PCR). Primer sets were used to amplify *CESA1, CESA3*, and *CESA6* (**Supplementary Table S1**). Unless otherwise stated the annealing temperature and time used were 55^o^C for 30 s with an extension for 1 min. The enzyme used was Ex Taq polymerase from TaKaRa. The PCR products were purified using the QIAquick PCR Purification Kit. Fifty nanograms of PCR product were placed in seven microliters of MilliQ water along with approximately 5 pmols of the forward or reverse primer in 0.7 μL. The samples were then sent to the Centre for Applied Genomics in Toronto, ON, Canada for direct PCR sequencing.

To genotype segregating F2 plants, we took advantage of differences in restriction fragment length polymorphisms (RFLPs) between wild type DNA and eight of the mutant plants (**Supplementary Table S2**). This was accomplished by selecting backcrossed F2 seedlings on 50 nM CBI that were either resistant or sensitive to the herbicide. These plants were transferred to soil and allowed to growth for 2 weeks before DNA was extracted and used for PCR amplification of CESA fragments using the primers listed in **Supplementary Table S1**. PCR products from at least 16 individuals from each population displaying either sensitive or resistant phenotypes were then digested with the restriction enzymes listed in **Supplementary Table S2**. To genotype mutant lines for which no RFLP marker existed, we collected 25 seedlings from a segregating F2 population, extracted DNA from the pooled seedlings and used it as template to amplify CESA gene fragments using the primers pair listed in **Supplementary Table S1**. PCR products amplified from DNA isolated from different seedling pools were then mixed in equal amounts to create either homoduplex or heteroduplex DNA by denaturing and cooling the DNA. Mixed products from resistant seedlings only formed homoduplex DNA, while products from resistant and sensitive seedlings formed heteroduplex DNA. The DNA was denatured by heating the mat to 95°C for 10 min and allowed to reanneal by slowly cooling the mixtures from 95°C to 25°C in 5°C decrements at a rate of -0.3°C and holding for 1 min at each step. These were analyzed using a mismatch-specific endonuclease (IDT DNA Technologies, Coralville, IA, United States) according to the manufacturer’s instructions.

### Phloroglucinol Staining of Lignin

Seeds were stratified for 4 days at 4^o^C in liquid 0.5× MS, then exposed to 200 μE/m^2^/s for 2 h at 20^o^C before being placed in the dark for 4 days. To visualize lignin, germinated seedlings were collected and stained with a solution of 3% (w/v) phloroglucinol in 95% (v/v) ethanol to which an equal volume of 50% (v/v) HCl was added. The stained seedlings were examined under a dissecting light microscope equipped with an AmScope MD900E camera (Irvine, CA, United States). The seedlings were examined for ectopic lignin staining and were compared against wild type controls (*n* = 20).

### ^14^C-Glucose Incorporation Into Cellulose

Five milligrams of homozygous seeds were weighed out on a microbalance in triplicate. Seeds were surface sterilized in a chlorine gas chamber and transferred to six-well plates containing 5 mL of 0.5× liquid MS medium (Sigma-Aldrich, St. Louis, MO, United States) supplemented with 0.5% (w/v) glucose then stratified at 4^o^C for 4 days. Plants were subsequently grown on an orbital shaker in the dark for 3 days. Seedlings were washed three times in a 5 mL glucose-free medium and suspended in 2 mL of 0.5× MS containing 0.5 μCi/mL ^14^C-glucose (American Radiolabeled chemicals, St-Louis, MO, United States) and incubated for 1 h in the dark on an orbital shaker. Following treatment, seedlings were washed three times with 5 mL of glucose-free medium, and then they were transferred to glass tubes and incubated in 5 mL of anhydrous ethanol at 80^o^C for 20 min. Seedlings were then incubated in 3 mL of chloroform: methanol (1:1) for 20 min at 45^o^C. Finally, seedlings were incubated in 5 mL of acetone at room temperature for 15 min. The acetone was aspirated and the tissue was allowed to dry completely before being weighed. Material was then treated with 400 μL of Updegraff solution (nitric acid: glacial acetic acid: water, 1:8:2) (Updegraff, 1969) in a boiling water bath for 1 h. Soluble and insoluble fractions were separated by passing the material through Whatman 25 mm GF/A glass microfilters. The flow-through was retained and represents the soluble fraction. The glass filters were subsequently washed six times with 4 mL of water and once with 4 mL of methanol, then dried in a 60°C oven for 2 h. Both soluble and insoluble fractions were transferred to separate scintillation vials, to which 5 mL of Ultima Gold High Flash Point Scintillation Liquid Cocktail (PerkinElmer, Waltham, MA, United States) was added. Beta emissions were quantified using a PerkinElmer Tri-Carb 2800 liquid scintillation detector (Waltham, MA, United States). Data is presented as percent incorporation acid insoluble material/(insoluble + soluble) × 100/mg dry weight.

### Powdered X-Ray Diffraction

Dried and processed senesced plant material was loaded onto an aluminum sample holder with a glass background. This, in turn, was placed into a PANalytical Phillips PW3170 X-ray diffractor. Material was pressed by hand using a scupula to generate an even surface. Protocols were run with a start angle of 2𝜃 = 4.5° and an end angle of 30°. The scan was run at a speed of 0.008° 2𝜃/s and at an intensity of 40 kV and 40 mA. Data was calculated using the equation for relative crystallinity index (RCI), where RCI = I_002_-I_am_/I_002_ × 100 (Segal et al., 1959). I_002_ is the maximal peak around 2𝜃 = 21.5^o^ for type I and 22.5^o^ for type II cellulose. I_am_ is the amorphous trough found around 2𝜃 = 18–20° (Segal et al., 1959). An average of 10 peaks were calculated at the amorphous trough and the crystalline peak areas. Reported results were from an *n* = 3, with each replicate containing plant material pooled from six individual plants.

### Acid and Enzymatic Hydrolysis of Plant Biomass

The senesced above ground tissue was ground to a powder using a Thomas Scientific Mill (model 3383-L10) and passed through a 60 gauge screen after being baked at 65^o^C for 24 h. Two hundred milligrams of dry tissue were placed in glass test tubes then filled with 10 mL of water. The tubes were vortexed and kept at room temperature overnight to allow the tissue to macerate. Once the majority of the tissue immersed to the bottom of the tube, the water was aspirated and a fresh 10 mL of water was added. The tubes were vigorously vortexed before being placed in an 80^o^C water bath for 1 h. The water was removed by aspiration and 10 mL of 70% ethanol was added to each tube, vortexed then placed at 80^o^C for 1 h. The ethanol was removed by aspiration and replaced with 5 mL of acetone. The tubes were vortexed and allowed to sit at room temperature for 15 min before the acetone was removed by aspiration. The tissue was allowed to dry for 2 days at room temperature. Ten milligrams of this tissue were placed in 1.5 mL screw-cap microcentrifuge tubes in triplicates.

#### Water Soluble Fraction

To each tube containing 10 mg of prepared tissue, 0.8 mL of water was added, the tubes were vortexed and kept at room temperature overnight. These were centrifuged at 14,000 *g* for 5 min and 50 μL of the supernatant was placed into separate wells of a 96-well spectrophotometric plate. One hundred microlitres of 0.2% anthrone in concentrated H_2_SO_4_ were added to the wells and mixed. The plate was placed on a heat block set at 100^o^C for 5 min then placed at 4^o^C for 10 min. Absorbance at 620 nm was determined using a BIO RAD xMark^TM^ Microplate Absorbance Spectrophotometer. The reducing sugar equivalents were determined by converting the absorbance readings to corresponding sugar quantities according to a glucose standard curve.

#### Acid Soluble Fraction

Fifty microliters of water were added to the tubes to replace the water removed in the previous assay. In addition, 0.2 mL of 1 M H_2_SO_4_ was added to bring the volume to 1 mL and the concentration to 0.2 M. These tubes were vortexed and kept at 80^o^C for 2 h. The tubes were allowed to cool to room temperature then centrifuged at 14,000 *g* for 5 min. Fifty microliters of the supernatant from each sample were assayed for their soluble reducing sugar content using 0.2% anthrone in the manner described above.

#### Enzyme Hydrolyzed Fraction

The dilute acid was aspirated and 1 mL of water was added to each tube. After vigorous vortexing, the tubes were centrifuged at 14,000 *g* for 5 min. The water was aspirated and replaced with 0.9 mL of 50 mM sodium citrate (pH = 4.8). Twenty microliters of 0.1× Celluclast (Sigma) and 80 μL of 0.1× Novozyme 188 (Sigma) were added to each tube to bring the final volume to 1 mL. The optimal activities of the enzymes were determined to be 111 FPU/mL and 500 U/mL, respectively (Ghose, 1987). After vortexing, the tubes were placed in a water bath kept at 50^o^C for 48 h. Periodical vortexing was done. The tubes were then centrifuged at 14,000 *g* for 5 min and 5 μL of the supernatant were placed into 45 μL of water in a 96-well spectrophotometric plate. The reducing sugars were quantified using 0.2% anthrone as described above.

#### Recalcitrant Cellulose Fraction

The supernatant was then removed by aspiration and replaced with 1 mL of Updegraff solution (Updegraff, 1969). The tubes were kept in a boiling hot water bath for 1 h, allowed to cool, and then centrifuged at 14,000 *g* for 5 min. The supernatant was removed by aspiration and replaced with 0.2 mL of 72% (v/v) H_2_SO_4_ and kept at room temperature for 1 h. After the entire pellet had dissolved, 0.8 mL of water was added and the tubes were vortexed. Five microliters of the supernatant were placed into 45 μL of water in a 96-well spectrophotometric plate and assayed with 100 μL of 0.2% anthrone.

## Results

### *ixr* and *fxr* Alleles Confer Varying Degrees of Herbicide Resistance

In an effort to gain a better understanding of the modes of action of both isoxaben and flupoxam, we isolated alleles that conferred isoxaben or flupoxam resistance from an ethylmethyl sulfonate (EMS) mutagenized Ler population. We reasoned that conducting a selection at a lower concentration of herbicide (20 nM) than previously reported (Heim et al., 1989) would allow us to isolate alleles of *CESA* genes. In addition, no known resistance alleles had been reported for flupoxam and we wished to determine if this herbicide has similar targets to isoxaben.

**FIGURE 1:**
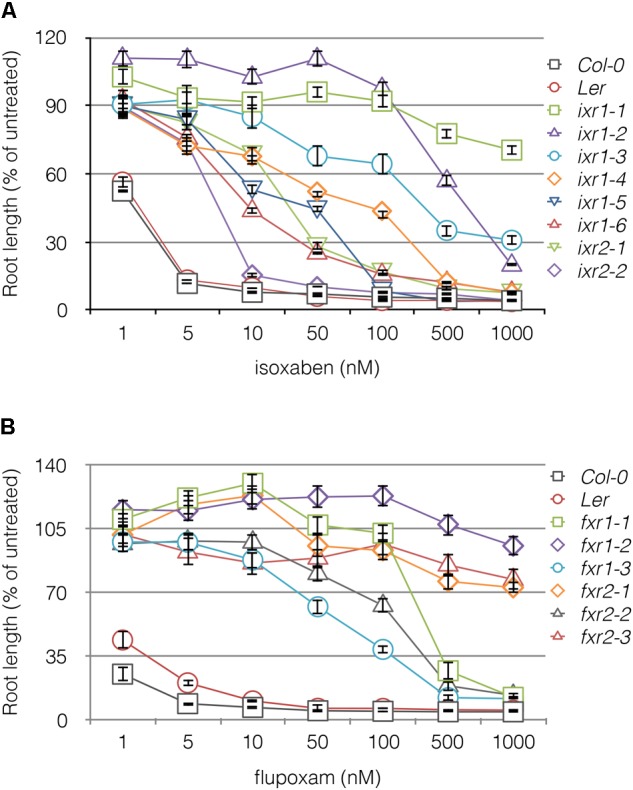
Resistance profiles of *ixr* and *fxr* mutants to isoxaben **(A)** and flupoxam **(B)**. The root lengths of 5 days old seedlings plotted against increasing concentrations of CBI. The vertical axis is expressed as a percentage of the root lengths on media containing no herbicide (untreated). Values are averages ± SD (*n* ≥ 20).

In total, we screened two million M2 seeds representing 50,000 M1 plants and isolated 12 mutant lines that were resistant to at least 20 nM of either herbicide. Five mutations were allelic to known isoxaben resistance (*ixr*) alleles (*ixr1-1, ixr1-2, ixr1-6, and ixr2-1*) (Scheible et al., 2001; Desprez et al., 2002; Sethaphong et al., 2013), while seven alleles conferred flupoxam resistance (*fxr*); none were cross resistant. At lower herbicide concentrations, 80% of the alleles conferred a higher percentage of resistance than would be expected for fully recessive alleles (**Supplementary Table S3**), which suggests that they are partially dominant at these lower concentrations. To distinguish primary binding sites from weak association with the destabilizing herbicide, the level of resistance of each mutant line was tested on varying concentrations of herbicide by measuring root lengths (**Figure 1**). Overall, *ixr* alleles confer a broader range of resistance to the herbicide compared with *fxr* alleles.

### Mutations in *ixr* and *fxr* Cluster in the C-Terminal Regions of CESA Proteins

**FIGURE 2:**
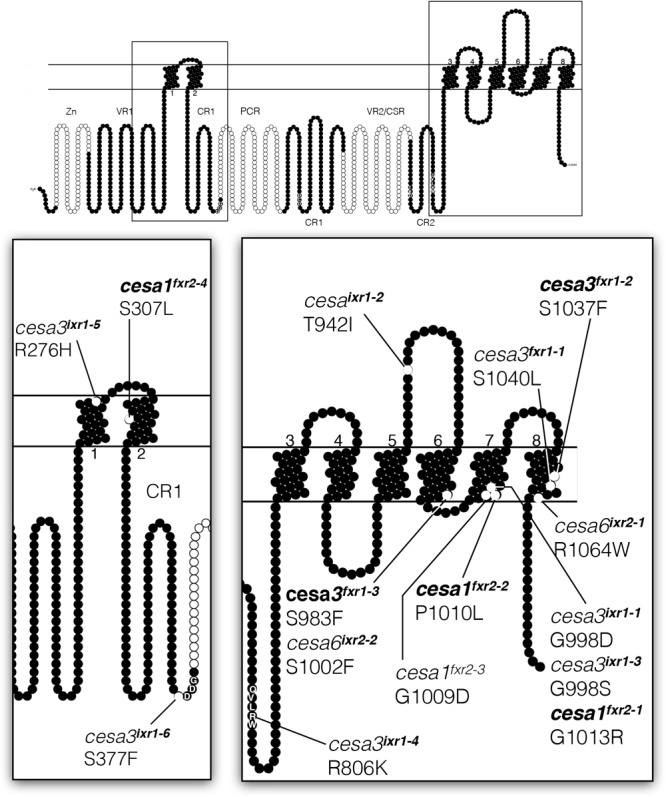
Diagram showing relative positions of *ixr* and *fxr* missense mutations on a generalized CESA protein. Structure is based on the CESA3 sequence and generated with Protter software (Omasits et al., 2014) with transmembrane topology prediction done with Phobius (Käll et al., 2004). The positions of CESA1 and CESA6 missense mutations are positioned at equivalent positions on the diagram based on sequence similarity alignments. Zn = zinc finger domain; VR = variable regions; CR/CSR = class specific regions; P-CR = plant-conserved region.

To determine the causal mutations conferring isoxaben resistance, we sequenced *CESA3* and *CESA6* genes from the mutant lines, knowing that other *ixr* alleles mapped to these genes. For flupoxam resistant mutants, we initially mapped three of the mutations using next generation mapping techniques (Austin et al., 2011) and subsequently sequenced primary cell wall *CESA* genes. All twelve lines harbored a single nucleotide polymorphism (SNP) in either *CESA1, CESA3*, or *CESA6*, with each of them resulting in single amino acid substitutions in the predicted protein sequence (**Figure 2** and **Supplementary Table S2**). One mutation, *fxr2-4*, was identified later than other mutations and was not phenotypically characterized but is included here for information. The resistance phenotype of segregating backcrossed F2 seedlings was correlated by making use of RFLP markers or, when RFLPs were not available, by DNA mismatch detection with a mismatch-specific endonuclease, CEL, which recognizes and cleaves mismatches resulting from SNPs (**Supplementary Table S2** and **Supplementary Figure S1**). Isoxaben resistance is conferred by alleles located in *CESA3* and *CESA6*, whereas flupoxam resistance is restricted to mutations located in *CESA1* and *CESA3*. In keeping with the nomenclature of existing mutations, we named *CESA3* alleles *ixr1* and *CESA6* alleles *ixr2.* Flupoxam resistant mutations were named so that *CESA3* alleles are *fxr1* and *CESA1* alleles are *fxr2*. An alignment of *Arabidopsis CESA* genes (**Supplementary Figure S2**) revealed that all of the mutations cause changes in invariant amino acid residues. Interestingly, in some instances equivalent amino acid residues were substituted in different *CESA* genes, which suggests that these sites are hotspots for conferring herbicide resistance. For example, a serine at position 983 in CESA1 is substituted with a phenylalanine in *fxr1-3;* the corresponding serine in CESA6 at position 1002 is also substituted with a phenylalanine in *ixr2-2.* Furthermore, in *fxr2-1* a glycine at position 1013 in CESA1 is substituted at an equivalent position in CESA3 (998) in both *ixr1-1* and *ixr1-3*. The majority (8 of 12) of the mutations are located in the C-terminal regions of the CESA proteins and in particular the last three being in the putative transmembrane domains. Three of the *fxr2* alleles map to the seventh putative transmembrane domain of CESA1, while *fxr2-4* maps to the second putative transmembrane domain. The *fxr1* alleles map to the sixth and eighth putative transmembrane domains of CESA3. In contrast, *ixr1* alleles are distributed throughout the CESA3 in both the catalytic region and in putative transmembrane regions. It is worth noting that the *ixr1, fxr1* or *fxr2* alleles causing the highest levels of resistance are all located in the C-terminal transmembrane regions of either CESA1 or CESA3; the only exception is *ixr1-2*, which maps to a loop between the putative fifth and sixth transmembrane domains. Low level resistance is associated with *ixr1* alleles that map to the catalytic region (*ixr1-4 or ixr1-6*) of CESA3, or to CESA6 (*ixr2*). This suggests that the primary sites of action of the two herbicides are in the putative transmembrane domains of CESA1 and CESA3. Interestingly, a previous study using the inhibitor, C17 (Hu et al., 2016), identified mutations with identical substitutions to *fxr1-2, fxr1-3, fxr2-1, fxr2-2*, and *fxr2-4* (bolded in **Figure 2**), indicating that C17 has a similar mode of action to flupoxam. Whether flupoxam has effects on mitochondrial function, like C17, has not been determined.

### A Subset of *ixr* and *fxr* Mutants Have Features Associated With Reduced Cell Wall Cellulose

Next, we assessed whether any of the alleles caused cellulose deficiencies that were severe enough to lead obvious morphological changes in the plants. We chose to assess hypocotyl length, ectopic lignin accumulation, and plant height and rosette diameter since these features are sometimes altered in cellulose-deficient mutants. We assumed that substitutions that conferred high levels of resistance but that showed few cellulose-deficiency-associated phenotypes would indicate sites required for herbicide binding, but that were not necessarily crucial for cellulose synthase activity or CSC function. Low-level resistance associated with significant cellulose-deficiency phenotypes would, on the other hand, be indicative of regions important for cellulose synthase activity or CSC function.

Ectopic lignin accumulation as a result of cellulose deficiency has been reported in *elp1/pom1* (Zhong et al., 2002), *eli1* (Cano-Delgado et al., 2003), *the1* (Hematy et al., 2007) as well as *CESA* deficient mutants (Rogers et al., 2005; Steinwand et al., 2014; Mélida et al., 2015). In addition, ectopic lignification is associated with cellulose reductions caused by exposure to CBI application (Bischoff et al., 2009). Although not all cellulose deficient mutants display ectopic lignification, the upregulation of lignin biosynthesis is likely a compensatory mechanism to maintain the structural integrity of the cell wall. Among the mutants in our collection, only *ixr1-4* and *ixr1-6* displayed ectopic lignin deposition in etiolated seedling hypocotyls (**Supplementary Figure S3**). This feature results in a cell wall cellulose content reduction, which is consistent with some of the other cellulose deficiency phenotypes of the mutants described below.

**FIGURE 3:**
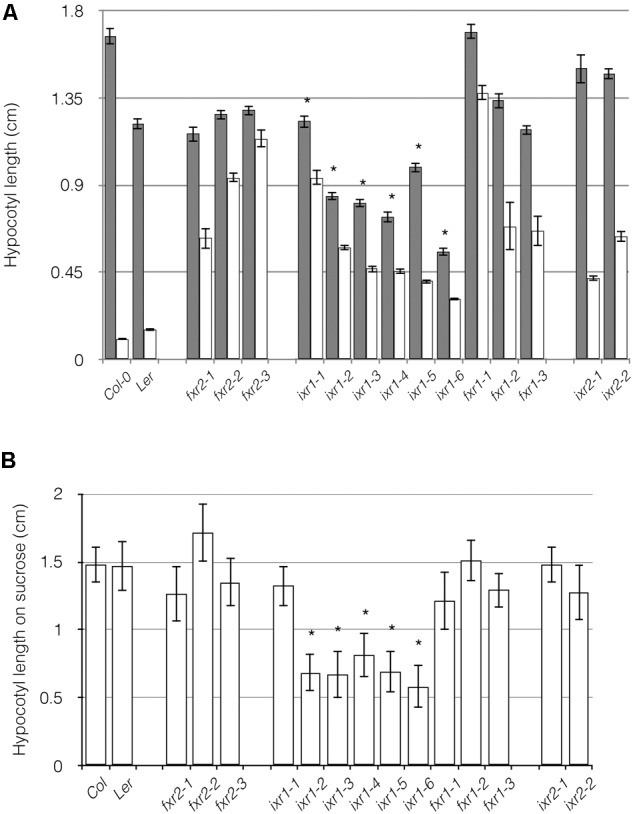
Comparison of hypocotyl lengths in wild-type and *ixr/fxr* plants. **(A)** The etiolated hypocotyl lengths of 8 days old seedlings grown in the absence (gray bars) and presence (white bars) of 10 nM of isoxaben or flupoxam is shown. All treated samples are significantly different from untreated samples (*P* ≤ 0.05). **(B)** Etiolated hypocotyl lengths of 8 days old seedlings grown on media containing 4.5% sucrose. Values are averages ± SD (*n* ≥ 20, ^∗^*P* ≤ 0.05 using Student’s *t*-test).

Hypocotyl cells elongate rapidly in the dark and as a consequence are sites of high rates of cellulose deposition. If cellulose deposition is dysfunctional, accelerated cell expansion is impeded, as it is sometimes evident in cellulose-deficient mutants (Fagard et al., 2000; Pagant et al., 2002; Refregier et al., 2004). Interestingly, only the *ixr1* alleles had shorter hypocotyls compared to wild-type controls (**Figure 3A**) indicating that these alleles affect CESA3 function under dark conditions. If seedlings were treated with CBIs, all seedlings had decreased hypocotyl lengths compared to -untreated seedlings (**Figure 3A**), indicating that the plants retain a conditional CBI sensitivity. This conditional sensitivity is consistent with the notion that CESA composition of the primary wall rosette is different or that it assumes altered conformational states in the light and the dark. Evidence supporting distinct genetic pathways modulating hypocotyl growth in dark- and light-grown *Arabidopsis* seedlings has been observed in mutant lines of *cesa6^procuste1^* (Desnos et al., 1996). The conditional cellulose deficiency of *cesa6^procuste1^* suggests that CESA6 is required for rapidly expanding cells such as in the hypocotyl under dark conditions. Our results imply that in addition to CESA6, CESA3 subunits might also be interchanged depending on the biological context.

A similar experiment involved in seedlings growth in the presence of high sucrose was conducted. The motivation for this, is that some cellulose defective mutants exhibit radial swelling in root cells (especially epidermal) on high sucrose (Benfey et al., 1993; Xu et al., 2008). When plants were grown in the dark on high sucrose, we observed that *ixr1* alleles had shortened hypocotyls (**Figure 3B**). However, *ixr1-4* and *ixr1-6* also had exaggerated the twisting of cell files around the long axis of the hypocotyl as well as being thickened (**Supplementary Figure S4A**). In addition, these same mutants had swollen roots when grown in the light (**Supplementary Figure S4B**) in a manner reminiscent of mutations leading to cellulose deficiencies (Xu et al., 2008).

**FIGURE 4:**
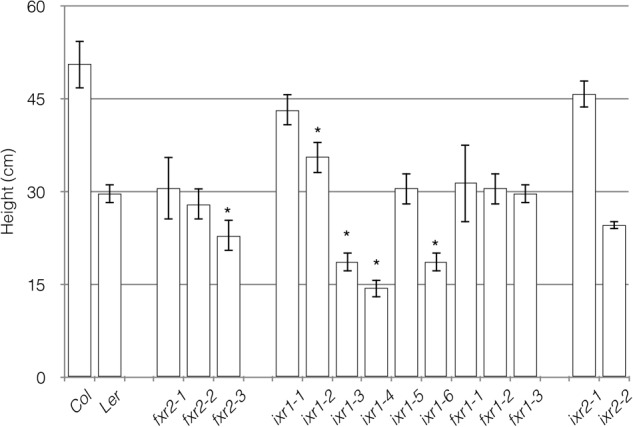
Average plant heights measured in mature wild-type and mutant plants. Values are averages ± SD (*n* = 6, ^∗^*P* ≤ 0.05 using Student’s *t*-test).

**FIGURE 5:**
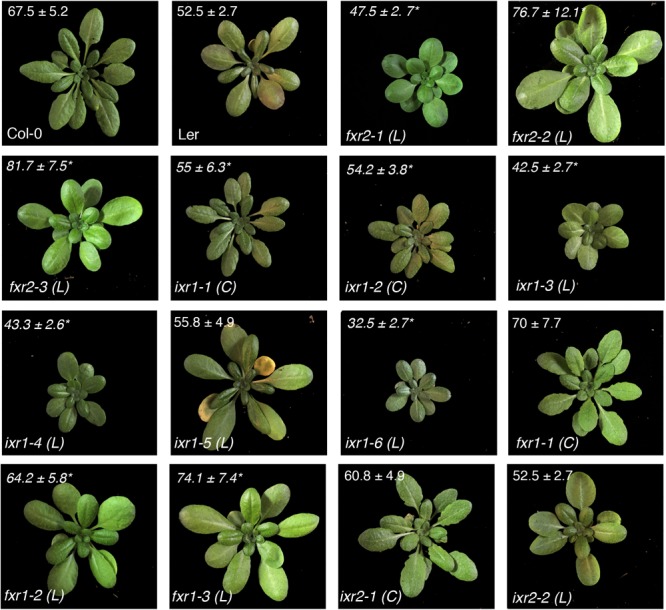
Representative appearance and size of rosettes. Rosette diameters (cm) at the onset of bolting measured as the distance from the edges of the rosette leaves are shown ± SD (*n* = 6, ^∗^*P* < 0.05 using Student’s *t*-test). Ecotype backgrounds for Col-0 (C) or Ler (L) are indicated next to mutant alleles.

Other growth parameters to assess potential differences among the mutants were overall plant height and vegetative rosette size. Most of the differences, although not exclusively, were observed in plants harboring *ixr1* mutations (**Figure 4**). These differences are consistent with the differences in hypocotyl length, although they are not unexpected for mutations mapping to the catalytic domain of the enzyme (e.g., *ixr1-4* and *ixr1-6*). The reduction in overall growth is also reflected in the size of vegetative rosettes. Again, most plants with reductions in rosette diameters were those harboring *ixr1* mutations (**Figure 5**). These observations suggest that while many of the mutations do not cause strong growth deficiency phenotypes, the CBIs, and in particular isoxaben, might act by disrupting CESA function in regions amenable to modification that are the least required for the catalytic activity of the enzyme.

### ^14^C-Glucose Incorporation Into Cellulose Is Affected by Some *ixr* and *fxr* Alleles

**FIGURE 6:**
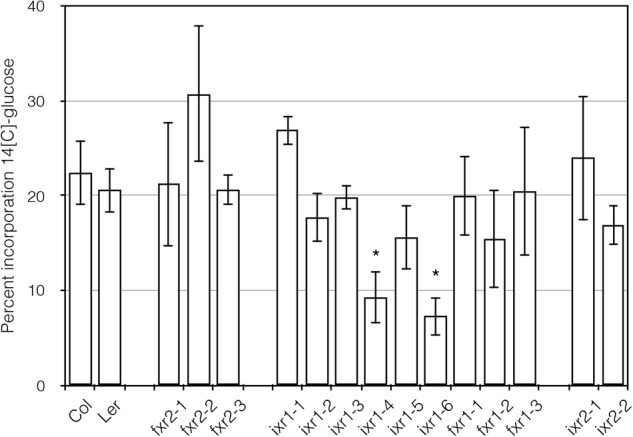
Glucose incorporation into the cellulose fraction of 3 days old etiolated seedlings. The vertical axis is expressed as the percentage of ^14^C-glucose measured in the acid insoluble fraction divided by the soluble plus insoluble fraction ± SD (*n* = 3, ^∗^*P* ≤ 0.05 using Student’s *t*-test).

Although direct measurement of CESA activity has proven difficult in plants, it is possible to obtain an approximation of this activity by conducting radioactive glucose incorporation, which measures the ability of the cellulose synthases to incorporate carbon-14 (^14^C) labeled glucose into cellulose. Again, we wished to determine lower incorporation rates when compared with CESA regions where herbicide action and catalytic activity overlapped. The incorporation results are expressed with respect to wild-type and is the ratio of the acid insoluble fraction and the total. Using this assay, two of the mutants showed decreases in glucose incorporation compared to wild type: *ixr1-4* and *ixr1-6* (**Figure 6**). It is not surprising that the mutants with decreased ^14^C-glucose incorporation, and thus an anticipated lower cellulose synthase activity, contain a substitution in the highly conserved QxxR region (*ixr1-4*) or next to a conserved aspartate (*ixr1-6*). These regions are thought to be required for catalytic activity for all type-II processive glycosyltransferases and have been implicated in glycan processing as the polymer emerges from the CESA complexes (Saxena et al., 2001). In contrast, the remaining mutants showed comparable levels of glucose incorporation (**Figure 6**). These results indicate that the bulk of *ixr* and *fxr* mutations affect a different aspect of CESA function, which in some cases lead to the differences in growth.

### The Relative Cellulose Crystallinity Index of *ixr* and *fxr* Plants Is Altered

**FIGURE 7:**
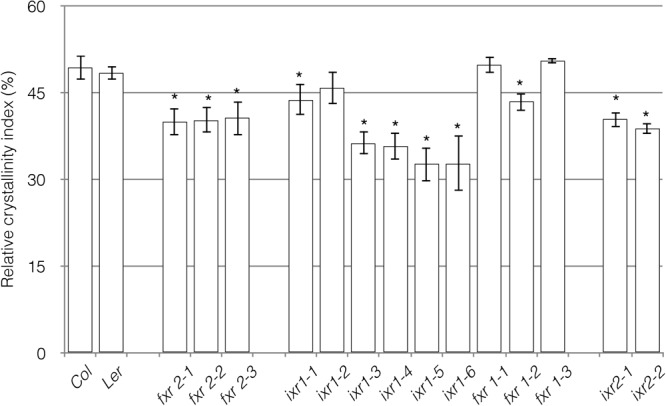
Relative crystallinity index (RCI) of powdered cell wall material. Measurements represent the RCI, calculated as I_002_–I_am_/I_002_ × 100 (Segal et al., 1959). I_002_ is the maximal peak around 2𝜃 = 21.5^o^ for type I and 22.5^o^ for type II cellulose. I_am_ is the amorphous trough found around 2𝜃 = 18–20°. An average of 10 peaks was calculated at the amorphous trough and the crystalline peak areas. Reported results are from an *n* = 3, with each replicate containing plant material pooled from six individual plants. Values are averages ± SD (^∗^*P* ≤ 0.05 using Student’s *t*-test).

Given the differences in plant growth, we wanted to examine if one of the consequences of the mutations would be on the final glucan product. More specifically, do the mutations affect the crystallinity of the cellulose embedded in the cell walls of the plants? A tool for determining cellulose structure is x-ray diffraction (XRD) (Segal et al., 1959; Harris and DeBolt, 2008). Based on the diffraction characteristics of two signature peaks, it is possible to calculate a RCI, which is, however, a relative measure of cellulose in the sample rather than an absolute measurement of cellulose crystallinity. To conduct these measurements, we used powdered above ground tissue of senesced plants. Overall, resistance to the CBIs appears to come at a cost to the RCI (**Figure 7**). Although *ixr1-2, fxr1-1*, and *fxr1-3* had RCI values similar to wild type, all of the other mutations lead to reduced RCI values. In general, the RCI inversely correlates with the severity of cellulose deficiency morphological phenotypes. The mutants with mild phenotypes show RCI values similar to wild type and those with more severe defects have significantly reduced RCI values compared to wild type. One of the underlying causes for morphological differences between the mutants and wild type might, therefore, be a reduction in cellulose crystallinity.

### Sequential Acid and Enzymatic Hydrolysis Reveals Enhanced Saccharification of *ixr* and *fxr* Cell Wall Material

**FIGURE 8:**
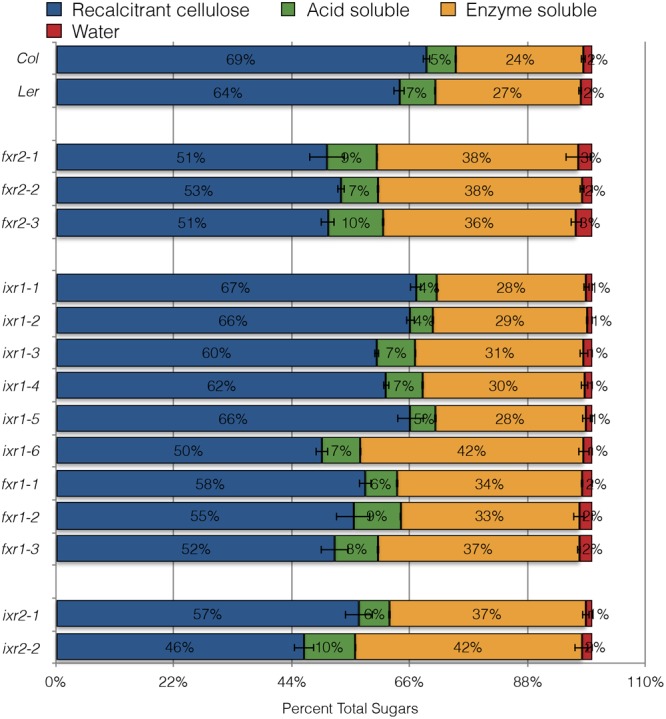
Sugar released from powdered, senesced tissue after sequential hydrolytic extractions. Sugar released during maceration in water (red), followed by extraction with 0.2 M acid (green), then with cellulase treatment (orange) and remaining cell wall material resistant to hydrolysis with nitric/acetic acid (blue). All values are averages ± SD (*n* = 3) and are expressed as a percentage of the total sugars released from cell wall material of each of the genotypes.

Cellulose crystallinity is considered to be a major factor contributing of biomass recalcitrance to hydrolysis. Generally, reduced crystallinity is attributed to allowing cellulases greater access and higher processing efficiency of the plant biomass (Harris et al., 2009). Since many of the mutants showed decreased crystallinity, their sensitivity to acid and enzymatic hydrolytic schemes were examined. To do this, we queried whether senesced-stem tissue of resistant mutants showed differences in hydrolysis sensitivity. The remaining inaccessible crystalline cellulose was quantified using a method described by Updegraff (Updegraff, 1969). The reducing sugar equivalents released at each step were quantified and expressed as the percentage of the total from all steps (**Figure 8**). While most of the cellulose remains inaccessible after the treatments, almost all of the CBI alleles show differences in cell wall accessibility. The greatest differences were observed after enzymatic hydrolysis with cellulase. It is intriguing that some of the alleles which showed differences in accessibility do not lead to growth defects (e.g., *fxr2* alleles). This is surprising since it would generally be assumed that modifications to CESAs resulting in a substantial decrease in cellulose RCI should be associated with plants having growth phenotypes. For example, the *ixr1-6* mutation leads to significantly increased biomass hydrolysis along with many plant growth defects. However, considering total sugar release per milligram of tissue, the *ixr2* and *fxr2* plants show a small number of cellulose deficiency phenotypes and only ∼50% total biomass remains recalcitrant to hydrolysis. While the correlation between enhanced saccharification and reduced cellulose crystallinity is consistent on the whole, in some cases (e.g., *fxr1-3*) some other yet unexplored difference seems to lead to enhanced saccharification.

## Discussion

Cellulose biosynthesis remains an incompletely understood process in plants. One of the main limitations has been that cellulose synthase activity has remained difficult to assay using *in vitro* techniques. We, therefore, used a forward genetic approach to identify mutations impinging on this process. Our approach took advantage of two known CBIs, isoxaben and flupoxam, which were originally developed as pre-emergence herbicides (Hoffman and Vaughn, 1996; Sabba and Vaughn, 1999; Vaughn and Turley, 2001; García-Angulo et al., 2012). In total, we isolated twelve alleles with increased resistance to either isoxaben or flupoxam. Our analysis indicates that the *CESA* genes, which encode cellulose synthases, are the primary targets of the two CBIs. In addition, our data indicate that certain amino acid residues are hotspots for herbicide resistance. For example, glycine-998 in CESA3 is the site of substitution for three alleles, *ixr1-1, ixr1-3*, and *fxr2-1*, suggesting that this glycine plays a key role for CBI resistance. The G1013R substitution in CESA1 (*fxr2-1*) is the equivalent residue of G998D and G998S in *cesa3^ixr1-1^* and *cesa3^ixr1-3^*, respectively. Furthermore, substitution of S1002F in CESA6 (*ixr2-2)* is at a corresponding residue S983F in *fxr1-3*. This, along with the similarity of effects that flupoxam and isoxaben exhibit, they also imply that the two CBIs have similar mechanisms of action. It is worth noting that the same residues that are substituted in *fxr1-2, fxr1-3, fxr2-1, fxr2-2*, and *fxr2-4* lead to resistance to another CBI, C17 (Hu et al., 2016). This feature further supports the notion that certain positions on CESA1 and CESA3 are hot spots for inhibitor binding and important for CESA function. Whether this involves the disruption of inter-CESA interactions or some other mechanism is not entirely clear, although treatment of plants with either isoxaben or C17, and presumably flupoxam, cause CSCs to become depleted from the plasma membrane, suggesting that they work by interrupting CESA interactions (Hu et al., 2016; Tateno et al., 2016). The deficiency phenotypes of some of the *ixr* and *fxr* mutants suggest that the inter-CESA binding could overlap with the herbicide binding. Considering that no CBI cross-resistance exists between *ixr* and *fxr* alleles and that all resistant lines map to primary cell wall *CESA* genes, one possibility is that both herbicides target two separate sites on the same complex. For example, they might disrupt primary wall rosette formation so that isoxaben targets CESA3-CESA6 interaction and flupoxam CESA3-CESA1 interaction. However, recycling of CSCs could equally be a result of subtle structural perturbations of the CESAs brought about by CBI binding.

The observation of non-polar to polar substitutions, conferring the greatest level of resistance, suggests that the CBIs act primarily within the membrane or at the cytosol-membrane interface. This is reasonable considering isoxaben and flupoxam are both relatively hydrophobic having log K_ow_ values of 3.94 and 3.27, respectively. Flupoxam resistance in CESA1 appears to be the greatest with the introduction of charge. For example, the *fxr2-2* (P1010L) mutation does not introduce a charge difference and does not lead to high levels of resistance like *fxr2-1* (G1013R) or *fxr2-3* (G1009D) (**Figure 2**). Another important finding is illustrated by comparing *ixr1-1* (G998D) and *ixr1-3* (G998S), which both have substitutions at the same site but incorporate different residues. Since glycine and proline are known to be important in determining tertiary structure, this suggests that their substitution leads to alterations of the herbicide binding site.

It is also interesting that the *fxr1* alleles, *fxr2-4, ixr1-6*, and *ixr2-2* are all predicted to cause replacements of serine residues. The significance of this bias is a matter for speculation. In the case of *ixr1-6*, the substituted serine for phenylalanine most likely disrupts the functional center of the protein. In other instances, it is possible that the residues are involved in inter-CESA interactions through hydrogen-bonding or are phosphorylation sites, which are known to affect CSC function (Taylor, 2007; Chen et al., 2010). However, there is no evidence to suggest that the serine residues substituted in *ixr* and *fxr* mutants are indeed phosphorylated (Nühse et al., 2004; Taylor, 2007; Chen et al., 2010; Jones et al., 2016), making this possibility less likely.

Overall, differences associated with the mutants highlight some interesting properties of the *ixr* and *fxr* mutants. High levels of resistance seem to favor amino acid substitutions predicted to be in the membrane interface. This is the case for *fxr1* and *fxr2* alleles, as well as for *ixr1-1, ixr1-*3, and *ixr1-5*. These alleles cause effects on the relative cellulose crystallinity index and enzymatic digestion of cell wall material, which is consistent with previous work indicating that the putative transmembrane regions of CESAs are required for cellulose crystallinity (Harris et al., 2012). A simple explanation for *ixr* and *fxr* resistance is that it is a result of slight conformational changes in the topology of the CBI binding site, reducing the affinity for the herbicide. Since many of the resistant mutants do not exhibit severe cellulose deficiencies, modification of the binding site must be possible without significant perturbation of the catalytic process. This implies that neither isoxaben nor flupoxam act primarily in the catalytic center and might instead have more of an effect on glucan extrusion. An exception to this trend is *ixr1-2* which leads to high isoxaben resistance but is located in a loop between two putative transmembrane regions. Its position is difficult to reconcile in relation to the other resistance alleles. However, this region is topologically ambiguous and aligns with a region of bacterial CESA that is between a cytosolic, interfacial helix and the putative seventh transmembrane of the BcsA protein (Morgan et al., 2013; Slabaugh et al., 2014a). It has therefore been speculated and evidence suggests that the loop is cytosolic rather than apoplastic (Slabaugh et al., 2014b), which would place the *ixr1*-2 site in closer proximity to the resistance hotspots.

In contrast, modifications at or near putative catalytic residues do occur but lead to low-level resistance. The R806K substitution in *ixr1-4* is within the highly conserved QxxRW domain and results in moderate resistance to isoxaben. This change is accompanied by phenotypes that are typically associated with cellulose deficiency like short stature, ectopic lignin accumulation, and reduced ^14^C-glucose incorporation. Similarly, *ixr1-6* leads low-level isoxaben resistance, cellulose deficiency phenotypes, and has a substitution (S377F) immediately upstream of a conserved catalytic aspartate. Since only a single substitution is required for resistance, it is unlikely that the clustering of the resistance alleles in this series, which define highly conserved regions of the CESA proteins, is indicative of multiple binding sites for the CBIs. These features might be reflective of an intimate association of these catalytic residues with the transmembrane pore in a manner that is analogous to bacterial CESA (Morgan et al., 2016) and raises the possibility that the mode of action of these CBIs is to disrupt this interaction.

## Author Contributions

IS, RL, JS, and DB conceived and designed the experiments and analyzed the data. IS, RL, ZK, and PS performed the experiments. JS, JN, and DB contributed reagents, materials, and analysis tools. IS, JS, and DB wrote the paper.

## Conflict of Interest Statement

The authors declare that the research was conducted in the absence of any commercial or financial relationships that could be construed as a potential conflict of interest.

## Supplementary Material

FIGURE S1 **(A)** Restriction length polymorphism (RFLP) analysis of CBI sensitive and CBI resistant plants from backcrossed F2 populations. Each panel shows the restriction pattern of PCR amplified DNA fragments covering the regions of the *CESA* genes where *ixr* or *fxr* alleles are located. Panels on the left are digests from DNA amplified from individual plants showing sensitivity to 50 nM CBI and panels on the right for plants showing a resistance phenotype. Fragments were amplified using PCR primers listed in **Supplementary Table S1** and expected fragment lengths resulting from digestion with the appropriate enzyme are listed in **Supplementary Table S2**. **(B)** DNA mismatch detection by CEL endonuclease digestion of PCR amplified fragments from DNA isolated from pools of 25 sensitive or 25 resistant seedling segregating in backcrossed F2 populations. Lane 1 is homoduplexed DNA from *fxr1-1* resistant seedlings, lane 2 is heteroduplexed DNA resulting from mixing *fxr1-1* resistant seedling with DNA from sensitive seedlings, lane 3 *fxr1-2* homoduplex, lane 4 *fxr1-2* heteroduplex, lane 5 *fxr2-1* homoduplex, lane 6 *fxr2-1* heteroduplex, lane 7 *ixr1-5* homoduplex, and lane 8 *ixr1-5* heteroduplex DNA. Arrows indicate the presence of bands resulting from CEL digestion of heteroduplex DNA which are absent in the homoduplex DNA controls, indicating that DNA from resistant seedlings contain the mutation. Molecular weight marker is lambda DNA digested with BstEI.

FIGURE S2 Local alignments of *Arabidopsis* CESA proteins. Locations of *ixr* and *fxr* missense mutations are indicated (*cesa1* mutations in green, *cesa3* mutations in red, and *cesa6* mutations in purple). Predicted transmembrane domains are underlined and conserved catalytic residues are bolded. Alignments were generated using ClustalW sequence alignment software (EMBL-EBI).

FIGURE S3 Representative lignin staining of dark grown hypocotyls of wild type and mutant plants. Only mutants that showed obvious ectopic lignin accumulation are shown. Bar = 250 μm.

FIGURE S4 Representative images of seedling hypocotyls **(A)** and seedling roots **(B)** from plants grown on media containing 4.5% sucrose. Compared to wild type plants, *ixr1-4* and *ixr1-6* plants show distinct morphological abnormalities. In hypocotyls, the mutants show clear torsion of cell files along the long axis. In roots, the epidermal cells of the mutants are disorganized and display isotropic growth. Bars represent 1 mm.

TABLE S1 A list of PCR primers used to amplify CESA regions used for sequencing and for RFLP analysis. Primers used to amplify the regions containing the SNPs for each mutant. The primers amplify approximately 600–1000 bp region containing the mutation.

TABLE S2 Segregation of resistance phenotype in backcrossed F2 *ixr* and *fxr* plants. Chi squared values are calculated for an expected recessive resistance trait. Values in red are not significant, while those in blue are significant and indicate deviation from the expected ratios.

TABLE S3 List of isoxaben and flupoxam resistant alleles and corresponding RFLPs. Four mutants in the table were previously described: *ixr1-1* (Heim et al., 1989), *ixr1-2* (Heim et al., 1990; Scheible et al., 2001), *ixr1-6* (Sethaphong et al., 2013), and *ixr2-1* (Desprez et al., 2002).
