# Supplementary figures and images for: Alleles Causing Resistance to Isoxaben and Flupoxam Highlight the Significance of Transmembrane Domains for CESA Protein Function
Source: Front Plant Sci. 2018 Aug 24;9:1152. doi: 10.3389/fpls.2018.01152 (PMC6118223; doi:10.3389/fpls.2018.01152)

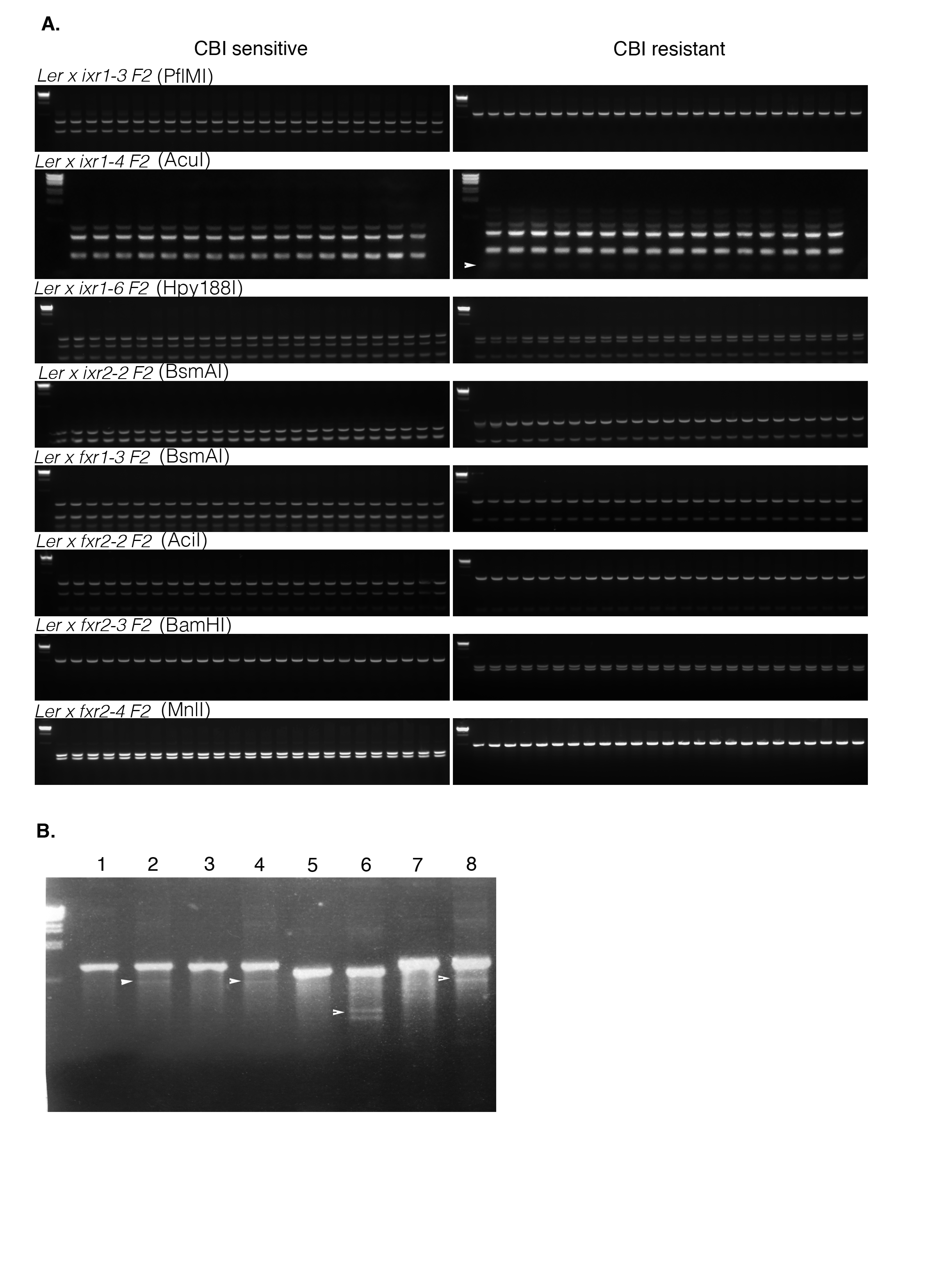

Supplement: FIGURE S1 — (A) Restriction length polymorphism (RFLP) analysis of CBI sensitive and CBI resistant plants from backcrossed F2 populations. Each panel shows the restriction pattern of PCR amplified DNA fragments covering the regions of the CESA genes where ixr or fxr alleles are located. Panels on the left are digests from DNA amplified from individual plants showing sensitivity to 50 nM CBI and panels on the right for plants showing a resistance phenotype. Fragments were amplified using PCR primers listed in Supplementary Table S1 and expected fragment lengths resulting from digestion with the appropriate enzyme are listed in Supplementary Table S2. (B) DNA mismatch detection by CEL endonuclease digestion of PCR amplified fragments from DNA isolated from pools of 25 sensitive or 25 resistant seedling segregating in backcrossed F2 populations. Lane 1 is homoduplexed DNA from fxr1-1 resistant seedlings, lane 2 is heteroduplexed DNA resulting from mixing fxr1-1 resistant seedling with DNA from sensitive seedlings, lane 3 fxr1-2 homoduplex, lane 4 fxr1-2 heteroduplex, lane 5 fxr2-1 homoduplex, lane 6 fxr2-1 heteroduplex, lane 7 ixr1-5 homoduplex, and lane 8 ixr1-5 heteroduplex DNA. Arrows indicate the presence of bands resulting from CEL digestion of heteroduplex DNA which are absent in the homoduplex DNA controls, indicating that DNA from resistant seedlings contain the mutation. Molecular weight marker is lambda DNA digested with BstEI. [file Image_1.TIF]

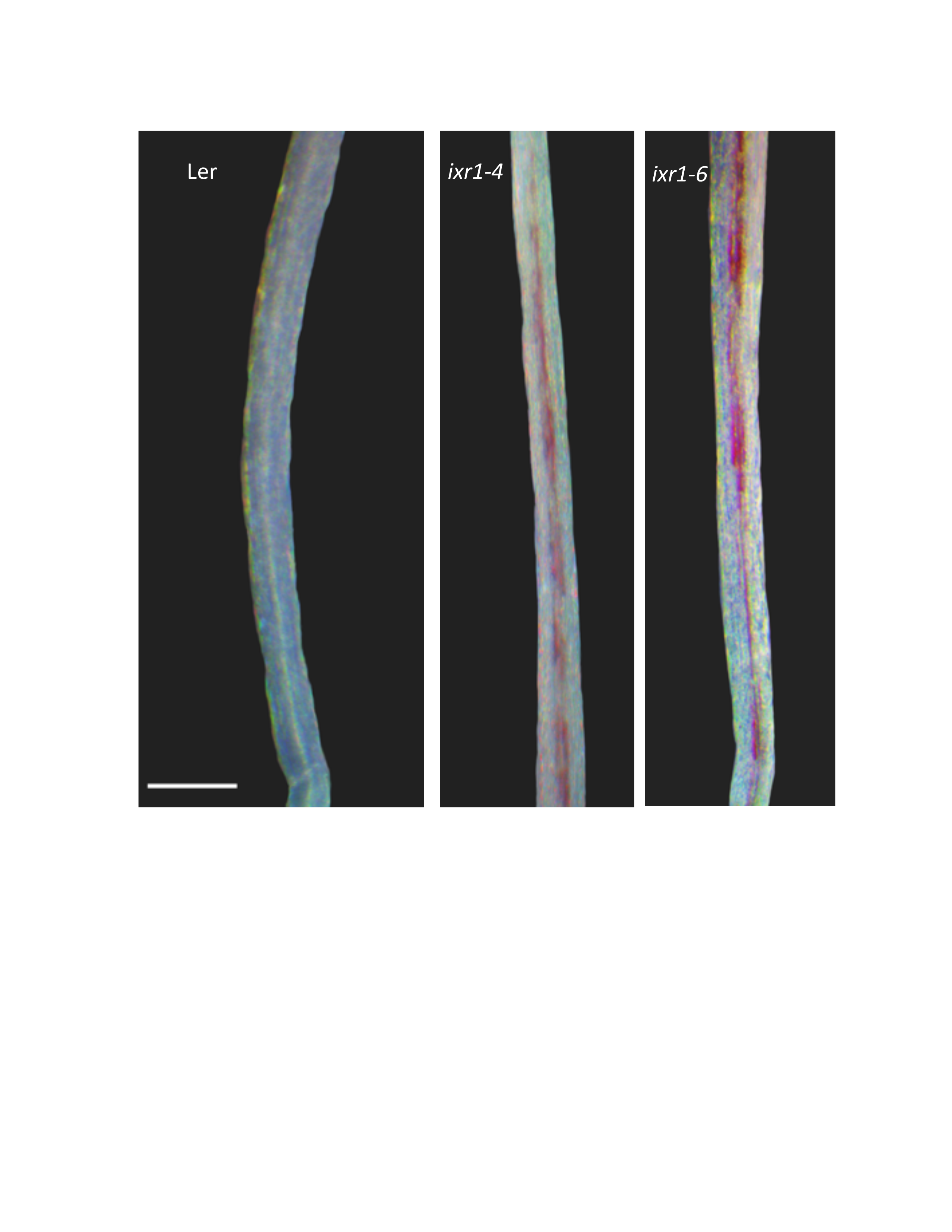

Supplement: FIGURE S3 — Representative lignin staining of dark grown hypocotyls of wild type and mutant plants. Only mutants that showed obvious ectopic lignin accumulation are shown. Bar = 250 μm. [file Image_3.tif]

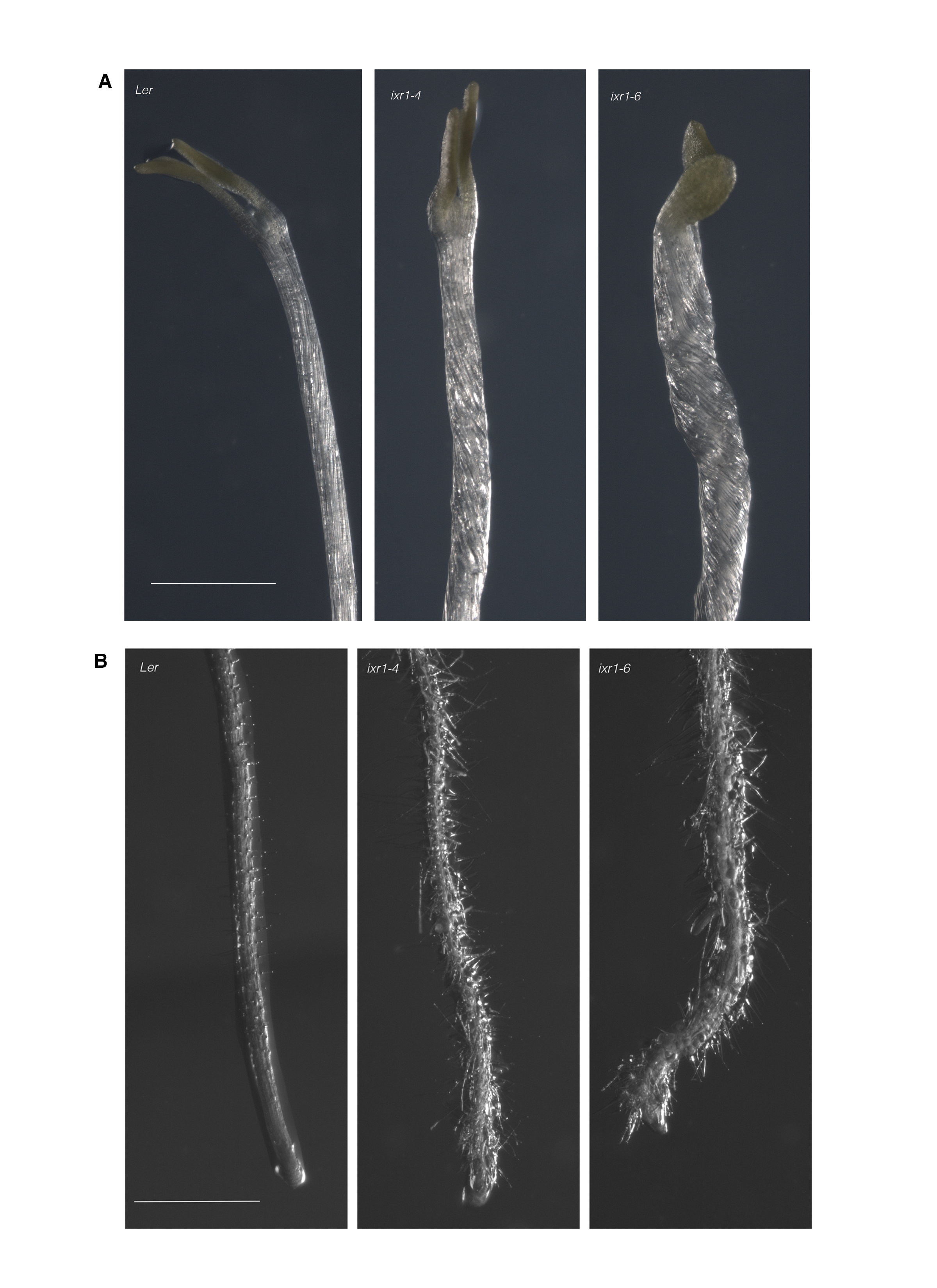

Supplement: FIGURE S4 — Representative images of seedling hypocotyls (A) and seedling roots (B) from plants grown on media containing 4.5% sucrose. Compared to wild type plants, ixr1-4 and ixr1-6 plants show distinct morphological abnormalities. In hypocotyls, the mutants show clear torsion of cell files along the long axis. In roots, the epidermal cells of the mutants are disorganized and display isotropic growth. Bars represent 1 mm. [file Image_4.tif]
