# Supplementary material for: Alleles Causing Resistance to Isoxaben and Flupoxam Highlight the Significance of Transmembrane Domains for CESA Protein Function
Source: Front Plant Sci. 2018 Aug 24;9:1152. doi: 10.3389/fpls.2018.01152 (PMC6118223; doi:10.3389/fpls.2018.01152)
Supplement: FIGURE S2 — Local alignments of Arabidopsis CESA proteins. Locations of ixr and fxr missense mutations are indicated (cesa1 mutations in green, cesa3 mutations in red, and cesa6 mutations in purple). Predicted transmembrane domains are underlined and conserved catalytic residues are bolded. Alignments were generated using ClustalW sequence alignment software (EMBL-EBI). [file Image_2.pdf]

```
                        cesa3ixr1-5        cesa1fxr2-4
                                 ↓                  ↓
CESA8    SYRIVIIMRLIILALFFNYRITHPVDSAYGLWLTSVICEIWFAVSWVLDQFPKWSPINRE  241
CESA5    PYRMLIVLRLVILGLFFHYRILHPVNDAYALWLISVICEIWFAVSWVLDQFPKWYPIERE  321
CESA6    PYRMLIVLRLVILGLFFHYRILHPVKDAYALWLISVICEIWFAVSWVLDQFPKWYPIERE  333
CESA2    PYRMLILCRLAILGLFFHYRILHPVNDAYGLWLTSVICEIWFAVSWILDQFPKWYPIERE  334
CESA9    PYRMLIFCRLAILGLFFHYRILHPVNDAFGLWLTSVICEIWFAVSWILDQFPKWYPIERE  339
CESA4    PYRIVIVLRLVILVFFFRFRILTPAKDAYPLWLISVICEIWFALSWILDQFPKWFPINRE  272
CESA1    PYRVVIILRLIILCFFLQYRTTHPVKNAYPLWLTLVICEIWFAFSWLLDQFPKWYPINRE  332
CES10    PYRIVIVLRLIILGVFLHYRTTHPVKDAYALWLTSVICEIWFAFSWLLDQFPKWYPINRE  319
CESA3    PYRMVIMLRLVILCLFLHYHITNPVPNAFALWLVSVICEIWFALSWILDQFPKWFPVNRE  316
CESA7    PYRMVIVARLVILAVFLRYRLLNPVHDALGLWLTSVICEIWFAVSWILDQFPKWFPIERE  295
          **::*. ** ** .*:.:*   *. .*  *** *********.**:******* *::**

         cesa3ixr1-6
         ↓
CESA8    SDDGAAMLSFESLVETADFARKWVPFCKKYSIEPRAPEFYFSLKIDYLRDKVQPSFVKER  361
CESA5    SDDGAAMLTFEALSETAEFARKWVPFCKKYTIEPRAPEWYFCHKMDYLKNKVHPAFVRER  441
CESA6    SDDGAAMLTFEALSETAEFARKWVPFCKKYCIEPRAPEWYFCHKMDYLKNKVHPAFVRER  453
CESA2    SDDGAAMLTFEALSDTAEFARKWVPFCKKFNIEPRAPEWYFSQKMDYLKNKVHPAFVRER  454
CESA9    SDDGAAMLTFEALSYTAEFARKWVPFCKKFSIEPRAPEWYFSQKMDYLKHKVDPAFVMER  459
CESA4    SDDGASMLLFDTLSETSEFARRWVPFCKKYNVEPRAPEFYFSEKIDYLKDKVQTTFVKDR  392
CESA1    SDDGSAMLTFESLSETAEFAKKWVPFCKKFNIEPRAPEFYFAQKIDYLKDKIQPSFVKER  452
CES10    SDDGSAMLTFEALSETAEFSKKWVPFCKKFNIEPRAPEFYFSQKIDYLKDKIQPSFVKER  439
CESA3    FDDGAAMLSFESLAETSEFARKWVPFCKKYSIEPRAPEWYFAAKIDYLKDKVQTSFVKDR  436
CESA7    SDDGASMLTFESLSETAEFARKWVPFCKKFSIEPRAPEMYFTLKVDYLQDKVHPTFVKER  415
         ****::** *::*  *::*:::*******: :****** **  *:***:.*:. :** :*

                                        cesa3ixr1-4
                                                ↓
CESA8    LTGFKMHCRGWRSIYCMPLRPAFKGSAPINLSDRLHQVLRWALGSVEIFLSRHCPLWYGC  744
CESA5    LTGFKMHSHGWRSVYCTPKIPAFKGSAPINLSDRLHQVLRWALGSVEIFLSRHCPIWYGY  831
CESA6    LTGFKMHSHGWRSVYCTPKLAAFKGSAPINLSDRLHQVLRWALGSVEIFLSRHCPIWYGY  846
CESA2    LTGFKMHCHGWRSVYCMPKRAAFKGSAPINLSDRLHQVLRWALGSVEIFLSRHCPIWYGY  845
CESA9    LTGFKMHCHGWRSVYCMPKRAAFKGSAPINLSDRLHQVLRWALGSVEIFLSRHCPIWYGY  849
CESA4    LTGFRMHCRGWKSVYCMPKRPAFKGSAPINLSDRLHQVLRWALGSVEIFFSRHCPLWYAW  809
CESA1    LTGFKMHARGWISIYCNPPRPAFKGSAPINLSDRLNQVLRWALGSIEILLSRHCPIWYGY  841
CES10    LTGFKMHARGWISIYCVPSRPAFKGSAPINLSDRLNQVLRWALGSIEILLSRHCPIWYGY  828
CESA3    LTGFKMHARGWRSIYCMPKLPAFKGSAPINLSDRLNQVLKWALGSVEILFSRHCPIWYGY  826
CESA7    LTGFKMHCRGWRSIYCMPKRPAFKGSAPINLSDRLNQVLRWALGSVEIFFSRHSPLWYGY  787
         ****:**.:** *:** *   *************:*********:**::***.*:**.

                                                            cesa3ixr1-2
                                                                 ↓
CESA8    SIILTSVLELRWSGVSIEDLWRNEQFWVIGGVSAHLFAVFQGFLKMLAGLDTNFTVTSKT  864
CESA5    SIAVTGILEMQWGKVGIDDWWRNEQFWVIGGVSAHLFALFQGLLKVLAGVETNFTVTSKA  950
CESA6    SIAITGILEMQWGKVGIDDWWRNEQFWVIGGVSAHLFALFQGLLKVLAGVDTNFTVTSKA  965
CESA2    SIAVTGILEMQWGGVGIDDWWRNEQFWVIGGASSHLFALFQGLLKVLAGVNTNFTVTSKA  964
CESA9    SIAVTGILEMQWGKIGIDDWWRNEQFWVIGGVSSHLFALFQGLLKVLAGVSTNFTVTSKA  968
CESA4    SIIATAILELRWSGVSINDLWRNEQFWVIGGVSAHLFAVFQGLLKVLFGVDTNFTVTSKG  928
CESA1    SIAVTGILELRWSGVSIEDWWRNEQFWVIGGTSAHLFAVFQGLLKVLAGIDTNFTVTSKA  960
CES10    SIYASAILELKWSDVALEDWWRNEQFWVIGGTSAHLFAVFQGLLKVFAGIDTNFTVTSKA  947
CESA3    SIFATGILEMRWSGVGIDEWWRNEQFWVIGGVSAHLFAVFQGILKVLAGIDTNFTVISKA  945
CESA7    SIIVTGILELRWSGVSIEEWWRNEQFWVIGGISAHLFAVVQGLLKILAGIDTNFTVTSKA  907
         **  :.:**::*. :.::: *********** *:****:.**:**:: *:.********
```

cesa6^{ixr2-2}/*cesa3*$^{fxr1-3}$ *cesa1*$^{fxr2-3}$ *cesa1*$^{fxr2-2}$

```
CESA8    A--DDLEFGELYIVKWTTLLIPPTSLLIINLVGVVAGFSDALNKGYEAWGPLFGKVFFAF   922
CESA5    A--DDGEFSELYIFKWTSLLIPPTTLLIINVIGVIVGISDAISNGYDSWGPLFGRLFFAF   1008
CESA6    A--DDGEFSDLYLFKWTSLLIPPMTLLIINVIGVIVGVFDAISNGYDSWGPLFGRLFFAL   1022
CESA9    A--DDGEFSELYIFKWTSLLIPPTTLLIINIVGVIVGVSDAINNGYDSWGPLFGRLFFAL   1026
CESA4    ASDEADEFGDLYLFKWTTLLIPPTTLIILNMVGVVAGVSDAINNGYGSWGPLFGKLFFAF   988
CESA1    -TDEDGDFAELYIFKWTALLIPPTTVLLVNLIGIVAGVSYAVNSGYQSWDLLFGKLFFAL   1019
CES10    -SDEDGDFAELYVFKWTSLLIPPTTILLVNLVGIVAGVSYAINSGYQSWGPLMGKLLFAF   1006
CESA3    -SDEDGDFAELYLFKWTTLLIPPTTLLIVNLVGVVAGVFYAINSGYQSWGPLFXKLFFAF   1004
CESA7    -TD-DDDFGELYAFKWTTLLIPPTTVLIINIVGVVAGISDAINNGYQSWGPLFGKLFFSF   965
                *.:** .***:***** :::::*::*::.*.* *:..** :****:*:::*::
```

cesa1^{fxr2-1} (R)/*cesa3*$^{ixr1-1}$ (D)$^{/ixr1-3}$ (R)

cesa6^{ixr2-1}

```
CESA8    WVILHLYPFLKGLMGRQNRTPTIVILWSILLASVFSLVWVRINPFVSKTDTTSLS---LN   979
CESA5    WVILHLYPFLKGLLGKQDRMPTIILVWSILLASILTLLWVRVNPFVAK-GGPILEICGLD   1067
CESA6    WVIIHLYPFLKGLLGKQDRMPTIIVVWSILLASILTLLWVWVNPFVAK-GGPVLEICGLN   1081
CESA9    WVIVHLYPFLKGLLGKQDRVPTIILVWSILLASILTLLWVRVNPFVSK-DGPVLEICGLD   1085
CESA4    WVIVHLYPFLKGLMGRQNRTPTIVVLWSILLASIFSLVWVRIDPFLPKQTGPLLKQCGVD   1048
CESA1    WVIAHLYPFLKGLLGRQNRTPTIVIVWSVLLASIFSLLWVRINPFVDANPNA-NNFNGKG   1078
CES10    WVVAHLYPFLKGLLGRQNRTPTIVIVWSALLASIFSLLWVRINPFVSTTGVMSNSFMGE-   1065
CESA3    WVIVHLYPFLKGLMGRQNRTPTIVVVWSVLLAFIFLLLWVRIDPFTSRVTGPDILECGIN   1064
CESA7    WVIVHLYPFLKGLMGRQNRTPTIVVIWSVLLASIFSLLWVRIDPFVLKTKGPDTSKCGIN   1025
         **: ********::*:*:: ***:::** ****:::*:***::**
```

cesa3^{fxr1-2} *cesa3*$^{fxr1-1}$
